# Moving beyond inquiry: a secondary qualitative analysis on promoting racial justice in clinical care

**DOI:** 10.1186/s12909-023-04131-5

**Published:** 2023-03-23

**Authors:** Baffour Kyerematen, Raquel Garcia, Joy Cox, Donna M. Zulman, Megha Shankar

**Affiliations:** 1 Department of Medicine, UC San Francisco, San Francisco, USA; 2 Division of Primary Care and Population Health, Stanford University School of Medicine, Palo Alto, USA; 3 Duke University School of Medicine, Durham, USA; 4 Rutgers New Jersey Medical School, Office of Primary Care and Community Initiatives, Newark, USA; 5 Veterans Affairs Palo Alto Health Care System, Center for Innovation to Implementation, Palo Alto, USA; 6 UC San Diego Department of Medicine, Division of General Internal Medicine, San Diego, USA

**Keywords:** Medical education, Anti-racism, Health equity, Health communication

## Abstract

**Background:**

Anti-Black racism is prevalent in medicine, and anti-racism training is needed in medical education. One such training is the Presence 5 for Racial Justice (P5RJ) Curriculum which covers evidence-based anti-racism communication strategies that promote health equity for Black patients. The P5RJ Curriculum was developed using feedback from clinicians and trainees with diversity, equity, and inclusion (DEI) experience. In this study, we identify themes in recommended anti-racism language and phrases that surveyed clinicians and trainees use to promote racial justice and health equity in clinical care for Black patients.

**Methods:**

Secondary analysis of survey responses to identify themes in qualitative data. Dataset: Survey responses of specific phrases for anti-racism communication based on P5RJ Curriculum feedback. Population studied: N = 50 respondents (27 clinicians, 17 medical trainees, 6 unreported) recruited through convenience sampling and listservs of clinicians with DEI experience. An inductive qualitative analysis was performed on survey responses to identify emerging themes.

**Results:**

Emerging themes from survey responses reflected four communication practices: “Inquiry” was the predominant practice (59%), followed by “Empathy” (25%), “Statements of Allyship” (9%), and “Self-Accountability” (8%).

**Conclusion:**

Inquiry and empathy may be predominant communication practices when addressing anti-Black racism in medicine. There is an opportunity to expand anti-racism communication tools with statements of self-accountability and allyship. Future research is necessary to analyze the patient voice on clinician communication practices that promote anti-racism in clinical care.

## Background

The recent re-focus on anti-Black racism in the United States has spurred anti-racism efforts across medical education [1]. While various curricula aim to foster anti-racism from the perspective of social determinants of health and/or diversity, equity, and inclusion [2], few provide trainees specific tools and language to address racism directly in the clinician-patient encounter [3]. There is a gap in the medical education literature in teaching trainees what to say in moments of racism to promote racial justice for Black patients. Similar to medical education trainings in how to deliver bad news [4], there is a need for training in how to address racism in the clinical encounter.

The Presence 5 for Racial Justice (P5RJ) Curriculum [5] leverages the Presence 5 framework [6] to promote racial justice in clinical training through evidence-based anti-racism communication strategies. In the development and refinement of the P5RJ Curriculum, we implemented an online survey for feedback from clinicians and medical trainees involved in diversity, equity, and inclusion (DEI) efforts. Part of our survey queried specific language and phrases that medical trainees and practicing clinicians are currently using to promote racial justice in the clinical encounter. This paper is a secondary analysis of the specific language and phrases provided through this section of the survey, and this project is aimed towards clinician educators and trainees.

In this study, we sought to elucidate themes in communication strategies that clinicians and trainees currently use to address anti-Black racism in clinical care.

## Methods

### Study design

This study is a secondary qualitative analysis of responses from an online, nationwide survey of medical trainees and practicing clinicians, administered between December 2020 and February 2021 during the development and evaluation of the P5RJ Curriculum. Participation was voluntary and written informed consent was obtained from all participants. There was no incentive for survey completion. The study was approved by the Stanford School of Medicine IRB.

### Survey development and distribution

The P5RJ Curriculum and survey is based on the Presence 5 framework for humanism in medicine and was designed by the Presence 5 for Racial Justice Research Team (including all authors) at Stanford University School of Medicine. The original Presence 5 framework was developed through a systematic literature review, interviews with patients and clinicians, and a Delphi panel. This framework was used to develop an anti-racism curriculum for medical education, the P5RJ Curriculum, which seeks to teach medical trainees evidence-based anti-racism communication strategies to promote health equity for Black patients. The P5RJ Curriculum was developed through literature review, online Qualtrics feedback survey, and structured feedback synthesis by the research team. The feedback survey queried clinicians and medical trainees with DEI experience including health disparities, health equity, anti-racism, medical education, and racial justice. Respondents were recruited through convenience sampling and email listservs (*N* = 52) of clinicians and medical trainees with experience in DEI; these listservs included chapters of national medical school diversity organizations (e.g., White Coats for Black Lives, American Medical Students Association, Student National Medical Association). Additionally, the survey was distributed to individual professional contacts (*N* = 123) who have involvement in DEI efforts and medical education. Survey participation was limited to medical trainees and clinicians. The P5RJ Curriculum was implemented in various medical education settings, including local academic institution and national conferences, and participants were not familiar with this curriculum.

Survey questions included feedback on the P5RJ strategies, examples, and suggestions for additional recommended language/phrases used for anti-racism communication in the clinical encounter. Participants of the survey were provided with the titles and descriptions of each of the P5RJ strategies: (1) Prepare with Intention, (2) Listen Intently and Completely, (3) Agree on What Matters Most, (4) Connect with the Patient’s Story, and (5) Explore Emotional Cues. Participants were asked to include free-text style responses of specific phrases or actions that they employ directly in their communications with patients as it pertains distinctly to the stated definition of each strategy, e.g. “What specific phrases or examples of language do you use with patients for this practice (Prepare with Intention)?” Respondents could submit more than one response per question, and each question was optional. Respondents were also queried for demographics, role/level of clinical practice, and DEI involvement.

### Secondary analysis

We performed a secondary analysis on survey participants’ qualitative feedback, focusing on specific phrases/language and actions used for each of the P5RJ strategy using an inductive qualitative analysis [7]. To develop the codebook, authors BK and RG (a medical student and pre-medical student at the time of the study) derived key terms from previous research in anti-racism communication [8] found in the survey responses. We then conducted a literature review in PubMed of these terms, such as “empathetic statements,” “allyship,” and “self-accountability.” Through snowball sampling from relevant articles in the initial literature search, we developed codebook definitions. The codebook was validated by JC, DZ, and MS (a qualitative researcher, physician-scientist, and physician-educator) through independent review and group discussion. Authors BK and RG independently and manually coded all responses using Microsoft Excel, then discussed the coding of each response until thematic saturation was reached according to the definitions from the literature. All responses were coded with one or more of the four codes.

## Results

Survey response rate was 39% for individual outreach; we are not able to calculate response rate from listserv outreach. A total of 53 responses of specific language/phrases were submitted by a total of 50 clinicians and medical trainees; some respondents provided more than one phrase. Table 1 shows participant demographics, where 54% of participants identified as clinicians/faculty, 94% of participants have DEI experience, and 20% of participants identified as Black or African American.

**Table 1:** Survey participant demographics

	Number (*N* = 50)	%
Level of Clinical Practice		
Clinicians/Faculty	27	54
Medical Trainees (e.g., medical students, residents, fellows)	17	34
- Medical Students	8	16
- Residents	7	14
- Fellows	2	4
No Response	6	12
Involvement in Diversity, Equity, Inclusion Efforts		
Active involvement in DEI efforts^a^	47	94
- Teaching/Curriculum Development	26	52
- Committee	24	48
- Leadership or Administration	21	42
- Student recruitment	21	42
- Mentorship	21	42
- Faculty hiring	16	32
- Research	16	32
- Community engagement/programming	15	30
- Educational Workshops	15	30
- Write In	3	6
No active involvement in DEI efforts	3	6
Race/Ethnicity		
Asian	9	18
Black or African American	10	20
Hispanic, Latinx, or Spanish Origin	4	8
White	20	40
Write In (Indo-Caribbean)	1	2
Multiple Selections^a^	6	12
Gender		
Woman	37	74
Man	13	26
Non-Binary	0	0

^a^Participants were asked to select all that apply

Table 2 shows participant examples of specific phrases/language used for anti-racism communication. Using the Presence 5 framework as a foundation for the curriculum and survey questions, our qualitative analysis revealed emerging themes that added to this framework with a focus on specific language/phrases used for anti-racism communication. Thematic analysis of the responses generated four emerging themes of communication practices that participants used to address anti-racism in clinical encounters: (1) inquiry, (2) empathy, (3) accountability, and/or (4) allyship (Table 2). Most examples provided were categorized as “inquiry” (*n* = 31) and “empathy” (*n* = 13), with the least number of examples provided categorized as “allyship” (*n* = 5) and “accountability” (*n* = 4).

**Table 2:** Specific phrases and actions that trainees and clinicians use to address racism in clinical encounters

Theme	Exemplary Quotes and Actions
Inquiry	· “What effect (if any) do you feel race has had on your health or your interactions with the health care system?” · “I know for many of my patients, racial bias is often felt in medical settings or other parts of their lives. I’m wondering if this is true for you, and if you’d be willing to talk a little about that? We find that these experiences have important impacts on your health. “ · “What is most bothersome to you or causing you the most stress currently (medical and non-medical)?” · “How have you experienced racism in medicine?”
Empathy	· “You seem sad, but maybe I am misinterpreting that. How are you feeling? · “I see that you’re upset by something just now, is that correct? Is it anger, or sadness?“ · “I might be very angry if that happened, is that what you feel, or is it something else?”
Allyship	· “Would you let me know if you feel you can’t speak up and I will speak up for you?” · “I want you to feel like you are an equal on our team.” · “[…] it is common for people of color to have mistrust in the medical system give the history of how people of color have been marginalized by the medical system.”
Accountability	· “I am sorry I made that assumption” · I tend to be actually somewhat informal with my patients, so it breaks down the hierarchy a bit · Combatting and dismantling anti-Black racism is NOT just about making people aware of how Blacks are routinely victimized and terrorized by White supremacy and anti-Black racism. · […]expose medical students to the LONG, RICH history of Black medical and scientific accomplishments. That is to say, ALL medical students should know about the numerous Black women and men who have made significant contributions to the field and its advancement.

## Discussion

In this secondary qualitative analysis of a nationwide online survey on anti-racism communication strategies, participants provided examples of specific phrases/language used to promote racial justice in the clinical encounter. Participants provided more examples that were categorized as inquiry and empathy compared to accountability and allyship.

One potential explanation for this finding from the literature is that conventional medical training emphasizes inquiry-based patient interviewing as one of the first skills to master in clinician-patient interactions [9]. Thus, clinicians and medical trainees may feel more comfortable with open-ended question-asking when the topic of medical racism arises during a patient interview. Empathetic statements were the second most common type of communication practice, which might reflect a focus on empathy in medical training when approaching sensitive subjects with patients [10]. All four themes were captured in the P5RJ framework, rooted in values of humanism in medicine and lending itself towards inquiry or empathy-based statements. Still, statements of accountability and allyship were still less frequent even considering that the curriculum offered recommended anti-racism communication practices of allyship and accountability, where allyship is defined as a practice which involves actively acknowledging one’s positions of privilege and also understanding how these inequities are upheld and reinforced [11]. For example, in the “Prepare” section of the curriculum, an example prompt asked the learner to “engage in open dialogue around power dynamics & addressing racism in clinical interactions,” while the “Listen” section asked clinicians to “acknowledge how biases in medical education may influence clinical learning & decisions.” Therefore, given the presence of all four communication types in the P5RJ framework prompts, this suggests that accountability and allyship statements may be used less regularly or with less familiarity than inquiry and empathy-based statements by the participants surveyed.

While both inquiry and empathy are central to trust-building and exploration of patient concerns, solely using empathy as a tool to display remorse of racist behaviors or attitudes is deficient in properly addressing the nuanced and intersectional feelings of racism [12]. Statements of allyship and accountability may be a way for clinicians and trainees to promote anti-racism, which requires an investment and commitment to re-evaluating power dynamics that lead to racism in clinical settings. These communication types may be especially impactful in the context of the patient-clinician relationship, which has historically been permeated by racism, inequitable power, and mistrust [13, 14]. Emerging evidence suggests that reducing patient mistrust as it relates to medical racism requires a more comprehensive approach to positively impact overall health outcomes [15]. Statements of accountability aim to communicate the role that medicine has played in anti-Black racism and Black patients’ mistrust of the medical system as a significant contributor to historical racism and modern-day racial inequities. Through this lens, accountability may serve as a bridge for clinicians to demonstrate allyship towards Black patients and a more personal commitment to advocating for anti-Black racism [15]. Although further research is needed to determine the impact of these communication styles on patient-clinician interactions, our analysis suggests that there may be an opportunity for DEI training to include specific statements and actions that clinicians can use to move beyond inquiry and empathy to convey accountability and allyship in clinical care. By broadening the typical scope of anti-racist behaviors and communication practices, clinicians can add stronger communication tools that may increase their connection with patients when addressing racist behaviors in medicine.

### Limitations and future work

There are three main limitations to this study. First, in this secondary analysis, we use a small sample of participant feedback on an anti-racism curriculum to understand what clinicians and trainees use in anti-racism communication, rather than having asked this directly. Further, there is a smaller number of trainees (*N* = 17) compared to faculty (*N* = 27). Despite this, we feel the qualitative nature of this study allows us to capture existing anti-racism communication practices by both trainees and faculty, as there is evidence that it is possible to reach thematic saturation in small studies [16]. Additionally, participants were shown evidence-based strategies included in the curriculum and had the opportunity to provide additional recommended phrases and specific language used to promote anti-racism in the clinical setting. Second, we note that 94% of participants were involved with DEI initiatives at their institutions, and this appropriately influenced the feedback provided to include anti-racism practices recommended by those with DEI experience. Future research may involve clinicians who are involved in medical education at large to better understand current barriers to implementing anti-racism curricula. Ongoing work from our team includes the implementation and evaluation of this curriculum for a broader audience. Third, we acknowledge that there is no one-size-fits-all approach to addressing interpersonal racism in medicine; future studies should include the patient perspective and communication practices will need to be tailored to individual patients and settings. Although additional training may help reduce racist behaviors in clinical settings, achieving interpersonal anti-racism in medicine will require efforts coupled with addressing structural racism in medicine.

## Conclusion

Anti-racism communication in medicine is urgently required in medical education. The present study suggests that practicing clinicians and trainees may be more familiar with inquiry and empathy-based communication than accountability and allyship to address racism in medicine. As emerging research demonstrates that empathy and inquiry alone may be insufficient for comprehensive anti-racism efforts, our findings suggest a critical opportunity to expand current medical education and DEI training, particularly when medical schools are undergoing significant curricular change, to include specific training on anti-racism communication. Further research is needed to study the impact and efficacy of these clinician communication practices, understand patient perspectives, and incorporate anti-racism into medical education competencies. As a first step, moving beyond empathy and inquiry towards a more comprehensive approach to anti-racism in medicine that includes accountability and allyship may be a step in the right direction towards combating anti-Black racism in medicine.

## Data Availability

The datasets generated and/or analyzed during the current study are not publicly available due to the original IRB approval not explicitly stating publicly available data including demographics but are available from the corresponding author on reasonable request.

## Abbreviations

DEI: Diversity, Equity, and Inclusion
P5RJ: Presence 5 for Racial Justice
